# Lipid-based insulin-resistance markers predict cardiovascular events in metabolic dysfunction associated steatotic liver disease

**DOI:** 10.1186/s12933-024-02263-6

**Published:** 2024-05-20

**Authors:** Alessandra Colantoni, Tommaso Bucci, Nicholas Cocomello, Francesco Angelico, Evaristo Ettorre, Daniele Pastori, Gregory Y.H. Lip, Maria Del Ben, Francesco Baratta

**Affiliations:** 1 Department of Clinical Internal, Anesthesiologic and Cardiovascular Sciences, Sapienza University of Rome, Rome, Italy; 2 Department of Human Anatomy, Histology, Forensic Medicine and Orthopedics, Sapienza University of Rome, Rome, Italy; 3 Liverpool Centre for Cardiovascular Science at University of Liverpool, Liverpool John Moores University and Liverpool and Heart and Chest Hospital, Liverpool, UK; 4 Department of General and Specialized Surgery, Sapienza University of Rome, Rome, Italy; 5 Department of Clinical Medicine, Danish Center for Health Services Research, Aalborg University, Aalborg, Denmark

**Keywords:** MASLD, Cardiovascular events, Insulin resistance, TyG index

## Abstract

**Background:**

Insulin resistance (IR) is the cornerstone of Metabolic Dysfunction Associated Steatotic Liver Disease (MASLD), pathophysiologically being the key link between MASLD, metabolic disorders, and cardiovascular (CV) diseases. There are no prospective studies comparing the predictive values of different markers of insulin resistance (IR) in identifying the presence of MASLD and the associated risk of cardiovascular events (CVEs).

**Methods:**

Post hoc analysis of the prospective Plinio Study, involving dysmetabolic patients evaluated for the presence of MASLD. The IR markers considered were Homeostatic Model Assessment for IR (HOMA-IR), Triglycerides-Glycemia (TyG) index, Triglycerides to High-Density Lipoprotein Cholesterol ratio (TG/HDL-C), Lipid Accumulation Product (LAP) and Visceral Adiposity Index (VAI). Receiver operative characteristic (ROC) analyses were performed to find the optimal cut-offs of each IR marker for detecting MASLD and predicting CVEs in MASLD patients. Logistic and Cox multivariable regression analyses were performed, after dichotomizing the IR markers based on the optimal cut-offs, to assess the factors independently associated with MASLD and the risk of CVEs.

**Results:**

The study included 772 patients (age 55.6 ± 12.1 years, 39.4% women), of whom 82.8% had MASLD. VAI (Area Under the Curve [AUC] 0.731), TyG Index (AUC 0.723), and TG/HDL-C ratio (AUC: 0.721) predicted MASLD but was greater with HOMA-IR (AUC: 0.792) and LAP (AUC: 0.787). After a median follow-up of 48.7 (25.4–75.8) months, 53 MASLD patients experienced CVEs (1.8%/year). TyG index (AUC: 0.630), LAP (AUC: 0.626), TG/HDL-C (AUC: 0.614), and VAI (AUC: 0.590) demonstrated comparable, modest predictive values in assessing the CVEs risk in MASLD patients.

**Conclusion:**

In dysmetabolic patients HOMA-IR and LAP showed the best accuracy in detecting MASLD. The possible use of lipid-based IR markers in stratifying the CV risk in patients with MASLD needs further validation in larger cohorts.

**Supplementary Information:**

The online version contains supplementary material available at 10.1186/s12933-024-02263-6.

## Introduction

Metabolic dysfunction associated steatotic liver disease (MASLD) is the new definition to identify liver steatosis disease associated with metabolic disorders in absence of alcohol abuse, hepatotropic viruses’ infection, iatrogenic causes, or genetic etiologies [1]. Recent data showed that the MASLD definition identifies an overlapping population with that identified by the non-alcoholic fatty liver disease (NAFLD) definition [2]. NAFLD/MASLD is the most important chronic liver disease worldwide [3] and is strongly associated with metabolic syndrome (MetS) and its features including abdominal obesity, atherogenic dyslipidemia, hypertension, and type 2 diabetes mellitus (T2DM) [4, 5].

Insulin resistance (IR) represents the key link between NAFLD/MASLD and MetS [6]. The fat accumulation in hepatocytes leads to chronic low-grade inflammation promoting IR [7]. In turn, IR alters the lipolysis and increases de-novo lipogenesis [8] perpetuating the hepatic lipid accumulation and worsening the inflammatory state with consequent hepatocyte damage and lastly fibrosis [9].

MASLD diagnosis requires the association of fatty liver evidence with at least one of the MetS criteria [1]. Each component of the MetS is an independent risk factor, and the combination of multiple MetS criteria leads to an exponentially increased risk of cardiovascular events (CVEs) [10, 11], such as myocardial infarction, stroke, arrhythmias, and death [9, 12, 13].

MASLD is underdiagnosed due to its asymptomatic nature; indeed, it typically manifests without notable symptoms in its early stages. In the absence of clinical manifestations, MASLD is often diagnosed as an incidental finding during unrelated medical evaluations, whereas the onset of CVEs or liver-related events represent the most frequent clinical complications. Hence, markers that can detect MASLD presence, or assist in the cardiovascular risk assessment, could represent useful tools for the clinical management of these patients [5, 14].

The HOMA-IR (*Homeostasis Model Assessment - Insulin Resistance*) is the most used index to diagnose insulin resistance [15, 16] but its strongest limitation is that it cannot be used in diabetic patients [17–19]. Others lipid-based IR markers, such as triglycerides-glycaemia (TyG) index and triglycerides to high-density lipoprotein cholesterol ratio (TG/HDL-C), have been proposed to identify patients at risk for T2DM [20] and to stratify the IR severity in patients with diabetes [21]. More recently, the LAP (Lipid Accumulation Product) index [22, 23] and VAI (Visceral Adiposity Index) [24] have also been demonstrated as accurate markers of IR.

The aim of this study was to evaluate the diagnostic value of different IR markers in detecting the presence of MASLD, and to assess the potential prognostic role of these markers in identifying MASLD patients at risk of CVEs.

## Methods

The study is a post hoc analysis of the prospective Plinio Study (Progression of Liver Damage and Cardiometabolic Disorders in Non-alcoholic Fatty Liver Disease: An Observational Cohort Study. ClinicalTrials.gov Identifier: NCT04036357) conducted in subjects with at least one cardiovascular risk factor of the following: arterial hypertension, overweight/obesity (BMI ≥ 25 kg/m^2^), type 2 diabetes, dyslipidemia, and metabolic syndrome (MetS). The study protocol has been previously described elsewhere [25]. Subjects who consented to blood sampling, had no data missing, and completed at least 6 months of follow-up were included in the analysis. Patients with liver steatosis not meeting MASLD criteria were excluded. Written consent was obtained from all subjects before the study, according to the ethical guidelines of the Declaration of Helsinki. The Ethics Committee of the Policlinic Umberto I Hospital of Rome (ref. n_2277/2011) approved the study. All authors had access to the study data and reviewed and approved the final manuscript.

### Clinical scores

TyG Index was calculated as follows:

$$Ln\left[\frac{{Triglycerides }\,({mg}/{dl}){ \times} Glycaemia\, (mg/dl)}{2}\right]$$

TG/HDL-C was calculated as follows:

$$\frac{Triglycerides\, (mg/dl) }{High \,Density\, Lipoprotein\, Cholesterol \,(mg/dl) }$$

HOMA-IR was calculated as follows:

$$\frac{\left[Glycaemia\, (mg/dl) {\times} Insulin \,(mU/l)\right] }{405}$$

LAP (*Lipid Accumulation Product*) was calculated as follows:

- For men:

$$\left(Waist\, circumference \,[cm] - 65\right) \times \left(Triglycerides\, [mmol/l]\right);$$

- For women:

$$\left(Waist\, circumference \,[cm] - 58\right) \times \left(Triglycerides\, [mmol/l]\right).$$

VAI (*Visceral Adiposity Index*) was calculated as follows:

- For men:

$$ \left[ {\frac{{Waist\, circumference\left( {cm} \right)}}{{\left\{ {39.68 + \left( {1.88 \times BMI\left( {\frac{{kg}}{{m^{2} }}} \right)} \right)} \right\}}}} \right] \times \left[ {\frac{{TG\left( {\frac{{mmol}}{l}} \right)}}{{1.03}}} \right] \times \left[ {\frac{{1.31}}{{HDL\left( {\frac{{mmol}}{l}} \right)}}} \right] $$

- For women:

$$ \left[ {\frac{{Waist\, circumference\left( {cm} \right)}}{{\left\{ {36.58 + \left( {1.89 \times BMI\left( {\frac{{kg}}{{m^{2} }}} \right)} \right)} \right\}}}} \right] \times \left[ {\frac{{TG\left( {\frac{{mmol}}{l}} \right)}}{{0.81}}} \right] \times \left[ {\frac{{1.52}}{{HDL\left( {\frac{{mmol}}{l}} \right)}}} \right] $$

FIB-4, a non-invasive marker of liver fibrosis, was calculated as follows:

$$\frac{Age\, [year] * AST\, [UI/L]}{Platelets\, [{\times}109/L] * \surd \left(ALT \,[UI/L]\right)}$$

Fib4 was defined as low if < 1.3 (in patients aged less than 65 years) or < 2.0 (in patients with 65 years or more), and high if > 2.67 (independently from age) [25–27]. A low Fib4 rules-out the presence of advanced fibrosis while patients with high Fib4 are likely to have advanced fibrosis.

MetS [28], arterial hypertension [29], and diabetes [30] were defined according to the most recent international guidelines.

### Follow-up

During the follow-up data on CVEs were prospectively collected. Patients underwent periodical phone calls (every six months) and visits (every 12 months) in the outpatient clinic. Only the first CVE registered during follow-up was used in the analysis. CVE was confirmed by medical records (imaging or discharge letter). In case of a fatal event, information was obtained from relatives or general practitioners.

CVEs included a composite of ischemic stroke, myocardial infarction (MI), cardiac (stent or coronary artery bypass surgery), or peripheral arterial revascularization (carotid endarterectomy or lower limb percutaneous transluminal angioplasty), atrial fibrillation and cardiovascular death. Diagnosis of MI was made according to the definition proposed by the Joint ESC/ACCF/AHA/WHF Task Force [31]. Ischemic stroke was determined on clinical manifestations and confirmed by radiological findings according to the AHA/ASA guidelines [32] If a patient died within 4 weeks of MI or stroke, this event was recorded as fatal MI/stroke. Transient ischemic attack was defined according to the Classification of Cerebrovascular Diseases III [33]. Cardiovascular death included sudden death, progressive congestive heart failure, and procedure-related death. Death was classified as cardiovascular unless an unequivocal non-cardiovascular cause of death was recorded.

### Statistical analysis

Normally distributed variables were expressed as mean and standard deviation while non-normally distributed ones were expressed as median and interquartile range. Group comparisons were performed by unpaired Student’s t test and ANOVA test or by Mann-Whitney and Kruskal-Wallis when appropriate. Proportions and categorical variables were tested by the χ2 test.

Descriptive analyses were performed according to the presence of MASLD, and in patients with MASLD, according to the IR markers dichotomized based on the optimal-cut offs derived from the Receiver Operative Characteristic (ROC) analyses with Youden’s J statistic (J index). ROC curves were performed to find the optimal IR-markers cut-offs for MASLD detection, and only in MASLD patients, to identify those at high risk of cardiovascular events during the follow-up. Area under the curve (AUC) values were calculated using the method described by Delong et al. and compared among the three IR scores [34].

Multivariable logistic regression analyses were conducted to investigate the independent association between IR indexes, dichotomized based on the optimal cut-offs, and MASLD. The analyses were adjusted for age, sex, obesity, diabetes, arterial hypertension, previous CVE, and low Fib4.

The incidence rate of adverse outcomes was calculated as the number of events/total person-years ratio and reported as incidence for 100 persons - year with relative 95% Confidence Interval (95% CI). In patients with MASLD Kaplan-Meier curves with log-rank test were performed to investigate the association between dichotomized IR markers and the risk of CVEs.

Multivariable Cox regression analyses were performed to calculate the relative hazard ratios (HRs) and 95% CI for CVEs associated with each dichotomized IR marker. All Cox regression multivariable models were adjusted for age, sex, obesity, diabetes, hypertension, previous CVEs, and low Fib-4.

Additionally, we performed an interaction analysis to assess the risk of CVEs associated with each dichotomized IR marker in relevant subgroups based on the presence or absence of diabetes or a history of previous CVEs. All the interaction analyses were adjusted for the same variables used in the Cox-regression multivariable models.

All tests were two-tailed, and analyses were performed using computer software packages (SPSS- 27.0, SPSS Inc., and JMP software version 15-SAS Institute).

## Results

We included 772 dysmetabolic patients, among whom 85.5% (*n* = 660) were diagnosed with MASLD (age 55.6 ± 12.1 years, 39.4% women). MASLD patients had a higher prevalence of MetS, obesity, and diabetes, along with lower mean HDL-C levels and higher TyG levels compared to patients without MASLD. A non-statistically significant trend of higher prevalence of hypertension was found in MASLD patients (52.7% vs. 61.5%, *p* = 0.077) with no significant differences for the prevalence of previous CVEs, and mean Fib4 score between the two groups (Table 1).

**Table 1:** Population characteristics according to MASLD diagnosis

	No MASLD (*n* = 112)	MASLD (*n* = 660)	*P* value
Age, mean ± SD (years)	57.7 ± 13.8	55.2 ± 11.7	0.041
Women, n (%)	47 (42.0)	257 (38.9)	0.545
BMI, mean ± SD (kg/m^2^)	26.6 ± 3.9	30.3 ± 4.9	< 0.001
Obesity (BMI > 30 kg/m^2^), n (%)	23 (20.5)	315 (47.7)	< 0.001
Metabolic Syndrome, n (%)	26 (23.2)	398 (60.3)	< 0.001
Waist circumference, mean ± SD (cm)	96.1 ± 9.5	106.8 ± 11.8	< 0.001
Glycaemia (mg/dl), mean ± SD	97.1 ± 27.3	105.5 ± 28.5	0.004
Diabetes, n (%)	15 (13.4)	184 (27.9)	0.001
Triglycerides, median [IQR] (mg/dl)	96.0 [78.5-128.3]	136.0 [103.0-182.8]	< 0.001
HDL-C, mean ± SD (mg/dl)	56.3 ± 14.3	48.2 ± 13.5	< 0.001
Arterial hypertension, n (%)	59 (52.7)	406 (61.5)	0.077
Systolic BP, median [IQR] (mmHg)	120.0 [115.0-140.0]	130.0 [120.0-140.0]	0.017
Diastolic BP, median [IQR] (mmHg)	80.0 [70.0–80.0]	80.0 [70.0–85.0]	0.007
Previous CVEs, n (%)	8 (7.1)	34 (5.2)	0.390
AST, median [IQR] (UI/l)	19.0 [16.0–22.0]	21.0 [17.0–28.0]	< 0.001
ALT, median [IQR] (UI/l)	17.5 [14.0–23.0]	27.0 [19.0–42.0]	< 0.001
GGT, median [IQR] (UI/l)	17.0 [12.0–26.0]	26.0 [17.0–41.0]	< 0.001
Platelets, mean ± SD (x10^9^/l)	237.0 ± 64.2	238.2 ± 64.3	0.858
High Fib-4, n (%)	2 (1.8)	15 (2.3)	0.745
Low Fib-4, n (%)	88 (78.6)	539 (81.7)	0.438
HOMA-IR, median [IQR]	2.0 [1.4–2.8]	3.6 [2.6–5.6]	< 0.001
LAP, median [IQR]	36.6 [27.6–51.2]	68.7 [47.9–97.4]	< 0.001
VAI, median [IQR]	1.2 [0.9–1.7]	2.1 [1.4–3.3]	< 0.001
TyG index, median [IQR]	4.6 [4.4-4.7]	4.8 [4.6–4.9]	< 0.001
TG/HDL-C, median [IQR]	1.7 [1.2–2.5]	2.9 [2.0-4.4]	< 0.001

*ALT* Alanine aminotransferase, *AST* Aspartate aminotransferase, *BMI* Body mass index, *CVEs* Cardiovascular events, *GGT* Gamma-glutamyl transferase, *HDL-C* High-density lipoprotein cholesterol, *BP* Blood pressure, *LAP* Lipid accumulation product, *VAI* Visceral adiposity index, *TyG Index* Triglyceride-glucose index, *TG/HDL-C* Triglycerides to HDL-cholesterol ratio, *MASLD* Metabolic dysfunction-associated steatotic liver disease, *IQR* Interquartile range, *SD* Standard deviation

**Fig. 1:**
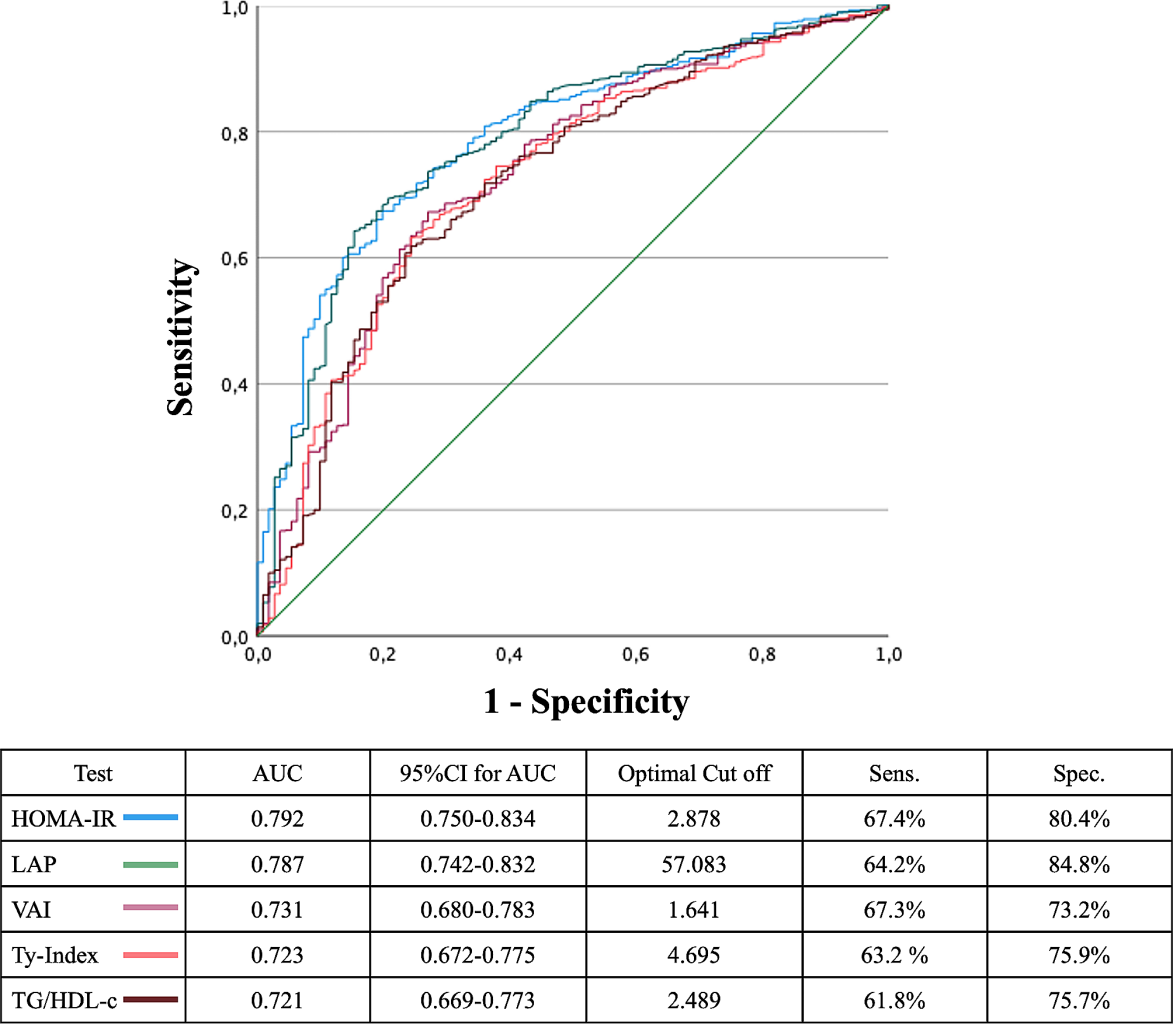
ROC curves of different insulin resistance markers in the screening of MASLD.

ROC analyses were conducted to determine the optimal cut-offs for the detection of MASLD. The AUC was calculated for each marker: for HOMA-IR 0.792 (95% CI 0.750–0.834); LAP 0.787 (95% CI 0.742–0.832), VAI 0.731 (95% CI 0.680–0.783), TyG index 0.723 (95% CI 0.672–0.775), and TG/HDL-C 0.721 (95% CI 0.669–0.773) (Fig. 1). When comparing the AUCs, the predictive value of HOMA-IR was significantly higher than VAI (*p* = 0.043), TyG index (*p* = 0.017), and TG/HDL-C (*p* = 0.019). Similarly, LAP had a significantly higher AUC than VAI (*p* < 0.001), TyG index (*p* < 0.001), and TG/HDL-C (*p* < 0.001). No significant differences were observed in the other AUC comparisons (Fig. 1).

The optimal cut-offs for MASLD detection were as follows: HOMA-IR 2.88 (sensitivity 67%; specificity 80%), LAP 57.08 (sensitivity 64%; specificity 85%), VAI 1.64 (sensitivity 67%; specificity 73%), TyG index 4.69 (sensitivity 63%; specificity 76%) and TG/HDL-C 2.49 (sensitivity 62%; specificity 76%).

To investigate factors associated with MASLD, we conducted a multivariable logistic regression analysis for each IR marker, categorizing them using ROC cut-offs. In these analyses, HOMA-IR ≥ 2.88 (Odds Ratio [OR] 6.33; 95% Confidence of Interval [CI] 3.78–10.61, *p* < 0.001), LAP ≥ 57.08 (OR 7.76; 95% CI 4.38–13.75, *p* < 0.001), VAI ≥ 1.64 (OR 4.51; 95% CI 2.84–7.17, *p* < 0.001), TyG index ≥ 4.69 (OR 4.26; 95% CI 2.64–6.88, *p* < 0.001), and TG/HDL-C ≥ 2.49 (OR 4.08; 95% CI 2.52–6.59, *p* < 0.001) were associated with MASLD independently from age, sex, obesity, diabetes, arterial hypertension, previous CVEs, and low Fib4 (Table 2).

**Table 2:** Multivariable logistic regression analyses of factors associated with MASLD

	Panel A	Panel B	Panel C	Panel D	Panel E
	aOR (95% CI)	aOR (95% CI)	aOR (95% CI)	aaOR (95% CI)	OR (95% CI)
Age	0.98 (0.96–1.00)*	0.98 (0.96–1.00)*	0.98 (0.96–1.00)	0.97 (0.95–0.99)**	0.98 (0.96–1.00)
Female sex	0.85 (0.54–1.35)	0.90 (0.57–1.42)	0.80 (0.51–1.25)	1.00 (0.64–1.57)	1.02 (0.65–1.61)
Obesity	2.12 (1.26–3.58)**	1.58 (0.92–2.71)	2.84 (1.71–4.72)***	2.78 (1.67–4.61)***	3.02 (1.80–5.04)***
Diabetes	1.81 (0.96–3.43)	2.19 (1.16–4.14)*	2.14 (1.15-4.00)*	1.80 (0.96–3.41)	2.27 (1.22–4.23)**
Arterial Hypertension	1.24 (0.77–2.01)	1.41 (0.87–2.29)	1.27 (0.78–2.04)	1.36 (0.84–2.20)	1.28 (0.79–2.08)
Previous CVEs	0.62 (0.24–1.61)	0.62 (0.24–1.57)	0.71 (0.28–1.79)	0.66 (0.26–1.68)	0.70 (0.28–1.75)
Low Fib-4	1.11 (0.64–1.93)	1.14 (0.66–1.99)	0.99 (0.57–1.70)	1.01 (0.60–1.74)	1.07 (0.62–1.84)
HOMA-IR ≥ 2.88	6.33 (3.78–10.61)***	–	–	–	–
LAP ≥ 57.08	–	7.76 (4.38–13.75)***	–	–	–
VAI ≥ 1.64	–	–	4.51 (2.84–7.17)***	–	–
TyG Index ≥ 4.69	–	–	–	4.26 (2.64–6.88)***	–
TG/HDL-C ≥ 2.49	–	–	–	–	4.08 (2.52–6.59)***

Panel A shows model including HOMA-IR ≥ 2.88, Panel B shows model including LAP ≥ 57.08, Panel C shows model including VAI ≥ 1.64, Panel D shows model including TyG Index ≥ 4.69, Panel E shows model including TG/HDL-C ≥ 2.49

*aOR* Adjusted odds ratio, C*I* Confidence interval, *CVEs* Cardiovascular events, *HDL-C* High-density lipoprotein cholesterol, *HOMA-IR* Homeostatic model assessment for insulin resistance, *LAP* Lipid accumulation product, *VAI* Visceral adiposity index, *TyG Index* Triglyceride-glucose index, *TG/HDL-C* Triglycerides to HDL-cholesterol ratio

*p < 0.05; **p < 0.01; ***p < 0.001

Given the lack of specificity of HOMA-IR in patients with diabetes, we conducted an interaction analysis to assess the robustness of the results obtained from the main analysis, stratifying by the presence or absence of diabetes (Supplementary Table 2). The risk of MASLD associated with each different IR marker above the optimal cut-off remained consistent regardless of diabetes status. While not statistically significant, there was a trend suggesting potentially lower performance of the TyG index in patients with diabetes (Diabetes: HR 1.83, 95% CI 0.54–5.72; No Diabetes: HR 5.30, 95% CI 3.04–9.24; p for interaction = 0.076).

### Follow-up

MASLD patients were followed for a median follow-up of 48.7 (interquartile range [IQR] 25.4–75.8) months, resulting in 2952 person-years of observation. During this period, 53 patients experienced CVEs, including 21 non-fatal MI, 6 ischemic non-fatal stroke/TIA, 11 peripheral arterial revascularizations, 7 incident atrial fibrillation, and 8 CV death. The annual incidence rate for CVEs was 1.8 (95% CI 1.4–2.4) per 100 persons-year.

**Fig. 2:**
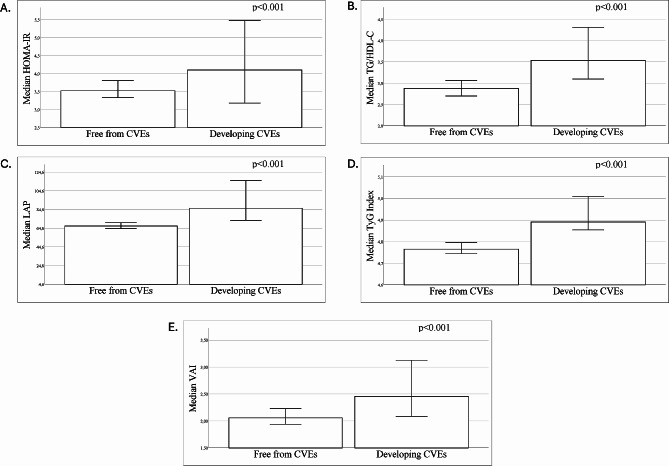
Baseline median values of different insulin resistance markers in patients with and without cardiovascular events during follow-up. HOMA-IR (**A**), TG/HDL-C (**B**), LAP (**C**), TyG index (**D**) and VAI (**E**). *CVEs *Cardiovascular events, *HDL-C* High density lipoprotein cholesterol, *HOMA-IR* Homeostatic model assessment for insulin resistance, *LAP* Lipid accumulation product, *MASLD* Metabolic dysfunction-associated steatotic liver disease, *TG* Triglycerides, *TyG Index* Triglyceride-Glucose Index, *VAI* Visceral Adiposity Index

IR markers, including TyG index (4.89 [4.71–5.07] vs. 4.76 [4.61–4.94], *p* = 0.002), LAP (86.01 [57.85–120.95] vs. 66.89 [47.30–94.43], *p* = 0.002), VAI (2.45 [1.83–3.63] vs. 2.06 [1.38–3.18], *p* = 0.029), and TG/HDL-C (3.54 [2.80–5.05] vs. 2.88 [1.96–4.39], *p* = 0.006) were higher in patients who developed CVEs during the follow up as compared to those who did not (Fig. 2). A non-statistically significant trend was observed for HOMA-IR (4.10 [2.92–6.42] vs. 3.52 [2.52–5.57, *p* = 0.080]) (Fig. 2).

**Fig. 3:**
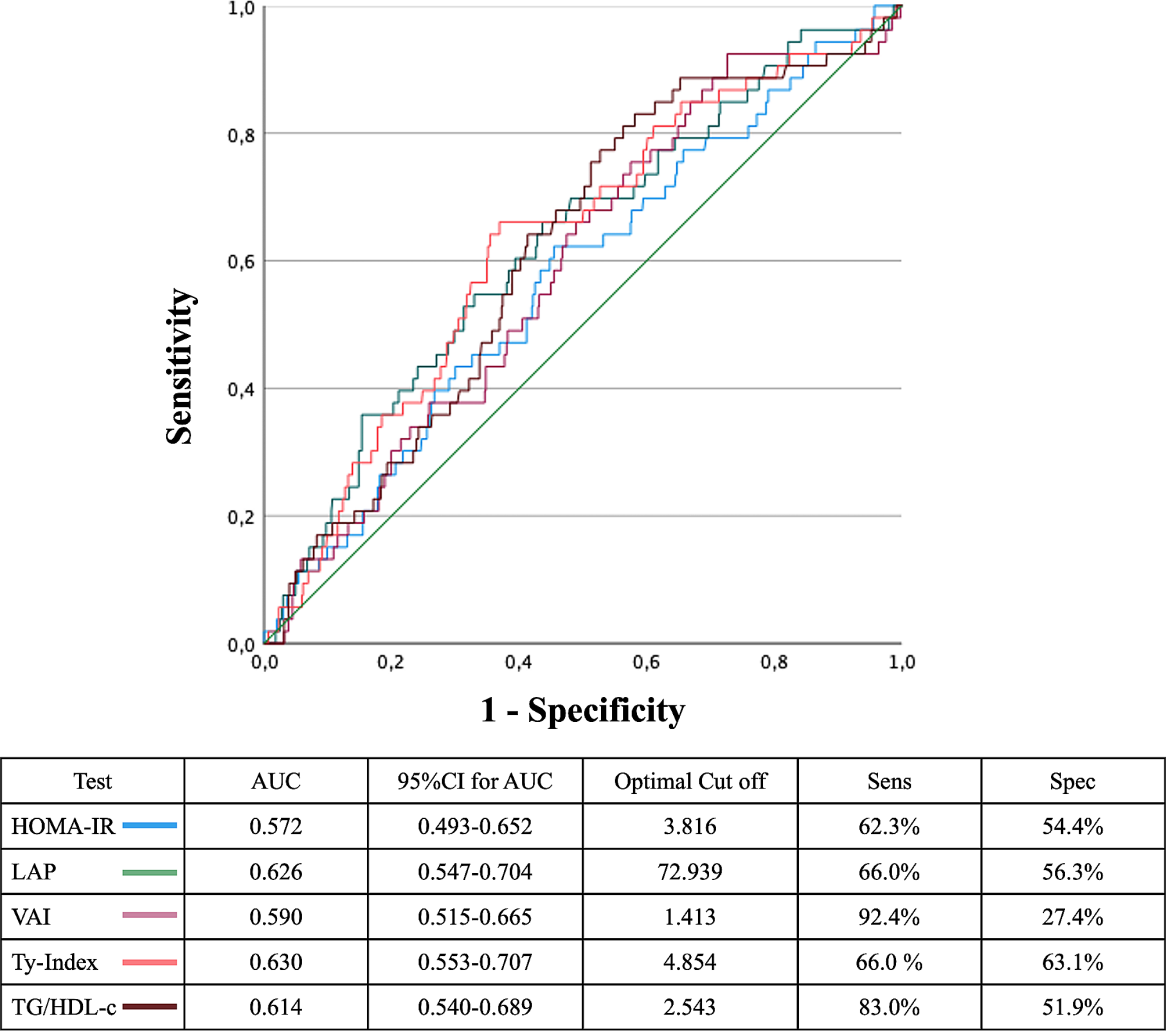
ROC curves of different insulin resistance markers in the detection of patients who develop CVEs

To identify the optimal indexes cut-offs for detecting patients who will develop CVEs we performed ROCs analyses. The AUCs were as follows: TyG index 0.630 (95% CI 0.553–0.707), LAP 0.626 (95% CI 0.547–0.704), TG/HDL-C 0.614 (95% CI 0.540–0.689), VAI 0.590 (95% CI 0.515–0.665), and HOMA-IR 0.572 (95% CI 0.493–0.652). No significant differences were found between the five ROC curves comparisons (Fig. 3). The best identified cut-offs were: TyG index 4.85 (sensitivity: 66%; specificity 63%), LAP 72.94 (sensitivity 66%; specificity: 56%), TG/HDL-C 2.54 (sensitivity 83%; specificity 52%), VAI 1.41 (sensitivity 92%; specificity 27%), and HOMA-IR 3.82 (sensitivity 62%; specificity 54%) (Fig. 3).

Clinical characteristics of MASLD patients according to the different cut-off for each IR markers are reported in Supplementary Table 1. Patients with IR indexes above the specific optimal cut-offs exhibited a higher prevalence of CV risk factors and a higher incidence of cardiovascular events during the follow-up, but no difference was found for the presence of liver fibrosis as assessed by Fib4.

**Fig. 4:**
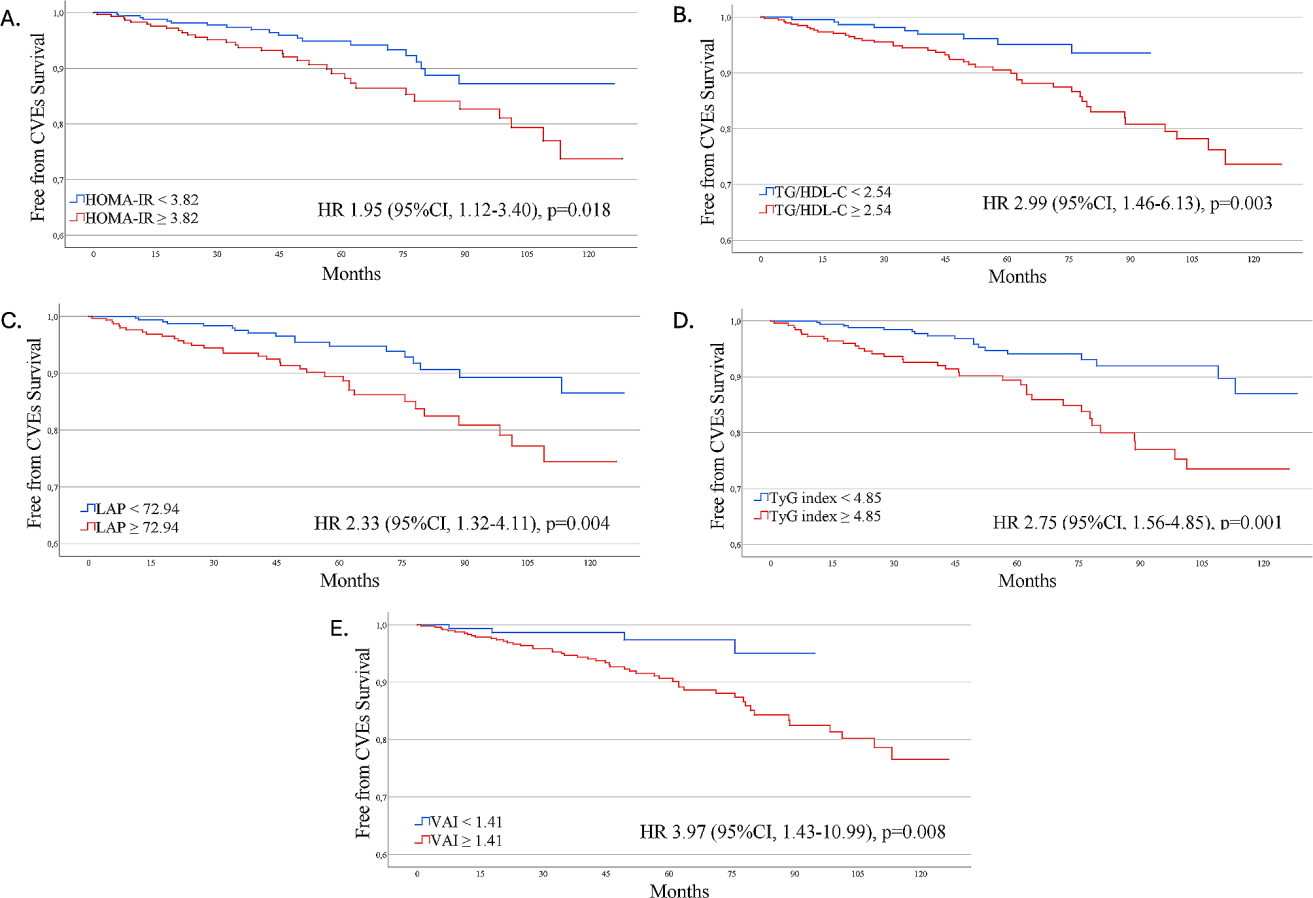
Kaplan Meier curves reporting CVEs-Free survival time according to the dichotomized insulin resistance markers. HOMA-IR (**A**), TG/HDL-C (**B**), LAP (**C**), TyG index (**D**) and VAI (**E**). *CVEs *Cardiovascular events, *HDL-C* High density lipoprotein cholesterol, *HOMA-IR* Homeostatic model assessment for insulin resistance, *LAP* Lipid accumulation product, *MASLD* Metabolic dysfunction-associated steatotic liver disease, *TG* Triglycerides, *TyG Index* Triglyceride-glucose index, *VAI* Visceral adiposity index

Kaplan–Meier analyses showed a significantly increased risk for CVEs among patients with TyG index ≥ 4.85 (*p* = 0.001), TG/HDL-C ≥ 2.54 (*p* = 0.003) LAP ≥ 72.94 (*p* = 0.004), VAI ≥ 1.41 (*p* = 0.008), and HOMA-IR ≥ 3.82 (*p* = 0.018) (Fig. 4). On Cox regression analysis, adjusted for confounders, the risk of CVEs remains significantly increased in patients with TyG index (aHR 2.44, 95% CI 1.35–4.14), LAP (aHR 2.33, 95% CI 1.28–4.25), TG/HDL-C ≥ 2.77 (aHR 2.85, 95% CI 1.37–5.92), and VAI (aHR 4.01, 95% CI 1.43–11.24) above the optimal cut-off (Table 3). No association between HOMA-IR and the risk of CVEs was found.

**Table 3:** Multivariable Cox regression analyses of factors associated with cardiovascular events in MASLD patients

	Panel A	Panel B	Panel C	Panel D	Panel E
	aHR (95% CI)	aHR (95% CI)	aHR (95% CI)	aHR (95% CI)	aHR (95% CI)
Age	1.04 (1.01–1.07)*	1.04 (1.01–1.07)**	1.04 (1.01–1.07)**	1.04 (1.01–1.07)**	1.04 (1.01–1.07)**
Female sex	0.41 (0.21–0.80)**	0.39 (0.20–0.76)**	0.39 (0.20–0.76)**	0.46 (0.23–0.90)*	0.46 (0.23–0.92)*
Obesity	1.42 (0.78–2.57)	1.32 (0.74–2.38)	1.51 (0.85–2.66)	1.56 (0.88–2.76)	1.57 (0.89–2.77)
Diabetes	1.09 (0.60–2.01)	1.29 (0.73–2.28)	1.28 (0.73–2.25)	1.09 (0.61–1.94)	1.23 (0.70–2.18)
Hypertension	1.08 (0.53–2.18)	1.33 (0.51–2.08)	1.00 (0.49–2.02)	0.97 (0.47–1.98)	1.00 (0.49–2.03)
Previous CVEs	3.00 (1.52-5,92)**	3.12 (1.58–6.14)***	2.81 (1.43–5.56)**	3.05 (1.54–6.05)***	2.94 (1.49–5.80)**
Low Fib-4	1.11 (0.57–2.16)	1.17 (0.60–2.28)	1.03 (0.53–1.98)	1.12 (0.58–2.17)	1.10 (0.57–2.13)
HOMA-IR ≥ 3.82	1.73 (0.93–3.21)	–	–	–	–
LAP ≥ 72.94	–	2.33 (1.28–4.25)**	–	–	–
VAI ≥ 1.41	–	–	4.01 (1.43–11.24)**	–	–
TyG Index ≥ 4.85	–	–	–	2.44 (1.35–4.41)**	–
TG/HDL-C ≥ 2.54	–	–	–	–	2.85 (1.37–5.92)***

All the multivariable models were adjusted for age, sex, obesity, diabetes, hypertension, previous CVEs, and Low Fib-4. Panel A shows model including HOMA-IR ≥ 3.82, Panel B shows model including LAP ≥ 72.94, Panel C shows model including VAI ≥ 1.41, Panel D shows model including TyG Index ≥ 4.85, and Panel E shows model including TG/HDL-C ≥ 2.54

*aHR* Adjusted hazard ratio, *CI* Confidence interval, *CVEs* Cardiovascular events, *HDL-C* High-Density lipoprotein cholesterol, *HOMA-IR* Homeostatic model assessment for insulin resistance, *LAP* Lipid accumulation product, *VAI* Visceral adiposity index, *TyG Index* Triglyceride-glucose index, *TG/HDL-C* Triglycerides to HDL-cholesterol ratio

*p < 0.05; **p < 0.01; ***p = 0.001

Considering the limited utility of HOMA-IR in diabetic individuals and potential biases associated with a history of CVEs, which could have influenced the risk of CVEs linked to IR markers, we conducted two separate interaction analyses stratified by the presence or absence of diabetes or a history of previous CVEs. Consistently with the main findings, these analyses revealed that the elevated risk of CVEs during the follow-up in patients with MASLD and lipid-based IR markers above the optimal cut-offs was irrespective of diabetes status and history of CVEs (Supplementary Table 3).

## Discussion

In this study, we assessed the diagnostic efficacy of five frequently used IR markers in detecting patients with MASLD, identifying HOMA-IR and LAP as the most effective indicators for this purpose. Second, our lipid-based IR indices—including the TyG index, TG/HDL-C ratio, LAP, and VAI—exhibit comparable predictive capabilities in identifying MASLD patients at increased risk of CVEs.

Our findings corroborate that all five IR markers have a moderate to high diagnostic value in detecting patients with MASLD, with the best accuracy for HOMA-IR and LAP, as confirmed by ROC analyses, with similar AUC to those previously reported [35, 36].

The association between HOMA-IR and NAFLD has been already reported in previous studies, which found a higher diagnostic value compared to other insulin resistance (IR) markers [37, 38]. Similar results were found for LAP; in a meta-analysis involving over 96,000 patients, the pooled sensitivity and specificity of LAP for screening NAFLD were 94% (95% CI 72–99%) and 85% (95% CI 62–96%), respectively [39].

We found IR markers cut-off for MASLD detection which differ from those previously reported. Isokuortti et al. found that in 368 non-diabetic patients, with a median HOMA-IR value of 1.6 [0.8–2.7], the optimal HOMA-IR cut-off for MASLD detection was 1.9 (AUC 0.85 [95% CI 0.80–0.89], sensitivity 80%, specificity 80%) [40]. Conversely, Gutierrez-Buey G et al. found that, in 57 diabetic patients, the optimal HOMA-IR cut-off for MASLD detection was 4.5 (AUC 0.81 [0.69–0.92], sensitivity 66%, specificity 93%) [41]. The discrepancy observed could be attributed to the different proportions of diabetic patients included in our study compared to those prior studies. In our population, we included both diabetic and non-diabetic patients, leading to a derived optimal cut-off for HOMA-IR of 2.8. This value encompasses the range reported in both diabetic and non-diabetic populations.

Comparing cut-offs for the TyG index among different studies is challenging due to variations in the formulas utilized. Previous studies have employed two different TyG index formulas, one including division by 2 in the logarithm argument and the other obtained through dividing the logarithm by 2. In our study, we utilized the original formula as reported by Simental-Mendía et al [42]. For this reason, the cut-offs obtained in our study cannot be directly compared with those previously published [43, 44]. Although using different formulas, Zou et al. [44] and Guo et al [43] found similarly consistent AUCs for the TyG index compared to those reported in our analysis (AUC 0.746 [95% CI 0.735–0.757] and AUC 0.761 [95% CI 0.747–0.774], respectively). Moreover, LAP and VAI were also evaluated in these studies. And were broadly consistent with our observations.

In the study performed by Zou et al., the optimal cut-off and AUC for MASLD detection utilizing LAP were 45.18 and 0.834 (95% CI 0.825–0.843), respectively; whereas Guo et al., found that the optimal cut-off was 28.72 with an AUC of 0.854 (95% CI 0.843–0.864). Conversely, for VAI, our findings (cut-off: 1.64, AUC 0.731 [95% CI 0.680–0.783]) are comparable to those previously reported, by Zou H. (cut-off: 1.494, AUC 0.741 [95% CI 0.730–0.752]) and Guo W. (cut-off: 1.426, AUC 0.773 [95% CI 0.759–0.786]).

Additionally, Fan et al. investigated the capacity of TG/HDL-C to detect NAFLD in 18,061 apparently healthy Chinese individuals [45]. TG/HDL-C was found to be independently associated with NAFLD, with different predictive values observed between women (cut-off: 0.9, AUC: 0.85 [95% CI 0.84–0.86]) and men (cut-off: 1.4, AUC: 0.79 [95% CI 0.78–0.80]). These data are in contrast with our results (cut-off: 2.49, AUC: 0.721 [95% CI 0.669–0.773]), but differences in ethnic origins (European and Asian) and the different prevalence of cardiovascular risk factors could provide some explanations.

The capacity of the lipid-related IR markers to predict CVEs across various clinical settings aligns with previous studies. Wang et al. reported an association between increased TyG index and the development of MACE over 3 years of follow-up in patients with diabetes and acute coronary syndrome [46]. Similar findings in non-diabetic patients are evident [47]; while Wan et al. reported a predictive role of TyG index in the general population free from previous CVEs [48]. In addition to its association with the risk of incident CVEs [49, 50], the TyG index has also been linked to poorer clinical outcomes following CVEs [47, 51–53].

Comparable results were reported for TG/HDL-C, which predicts worse outcomes in patients with acute coronary syndrome and ischemic stroke [54–60], with wide heterogeneity in its predictive value across different ethnicities [48]. Data regarding the relationship between LAP and VAI showed a significant association with both the short- and long-term risk of CVEs in non-obese patients [49–51]. This was further supported by the ATTICA study showing the predictive value of LAP for CV events over a 10-year period [61].

To date, research on the association between lipid-based IR markers and CVD in patients with NAFLD has primarily relied on cross-sectional and retrospective studies. Zhao et al. demonstrated that an elevated TyG index is correlated with the diagnosis and severity of coronary heart disease in a cross-sectional setting of NAFLD patients presenting with chest pain [62]. In another cross-sectional study of a large cohort of NAFLD patients, subclinical atherosclerosis has been linked to the TyG index [63]. An association between the TyG index and atrial fibrillation has also been noted [64]. Thus, our study is the first prospective cohort that showed the predictive value of lipid-based IR markers in identifying MASLD patients at risk of CVEs within a large observational prospective registry.

The prognostic value of lipid-based IR markers in predicting CVEs may be attributed to their calculation based on lipid parameters, which reflect the non-glycemic consequences of IR. Indeed, atherogenic dyslipidemia, characterized by low HDL-C and high triglyceridemia, which represents the typical lipid phenotype observed in insulin-resistant patients. This dyslipidemia pattern is primarily due to the increased secretion of VLDL [65] and decreased HDL efflux [66], contributing significantly to the heightened cardiovascular risk observed in this population [67–69].

### Strengths and limitations

This study has several strengths. We reported data on the association between lipid-based markers of IR in a Caucasian population, while most of the previous studies were conducted in Asians and Hispanics as previous described. In addition, we described the predictive role of IR markers in a prospective cohort of MASLD patients, confirming what was previously found in other clinical settings [35, 39, 70]. We also firstly described the predictive role of LAP and VAI index, usually less applicated in MASLD cohorts.

Our study has also some limitations. This study is a post-hoc analysis of a prospective study designed for other pre-specified outcomes. In addition, cross-sectional data on MASLD detection by IR markers were based on the US diagnosis of liver steatosis. However, although the US is not the gold standard for MASLD diagnosis, is the largest used technique for the clinical detection of fatty liver worldwide. Furthermore, certain potential confounding factors such as socioeconomic status, menopausal status in women, and the influence of various medical treatments were not considered in this analysis, which could introduce bias.

## Conclusions

In dysmetabolic patients HOMA-IR and LAP showed the best accuracy in detecting MASLD. The possible use of lipid-based IR markers in stratifying the CV risk in patients with MASLD needs further validation in larger cohorts.

### Electronic supplementary material

Below is the link to the electronic supplementary material.

Supplementary Material 1

## Data Availability

The datasets used and analyzed during the current study are available from the corresponding author upon reasonable request.

## Abbreviations

ALT: Alanine Aminotransferase
aOR: Adjusted Odds Ratio
AST: Aspartate Aminotransferase
AUC: Area Under The Curve
BMI: Body Mass Index
CHD: Coronary Heart Disease
CI: Confidence Interval
CVD: Cardiovascular Disease
CVEs: Cardiovascular Events
FIB-4: Fibrosis-4 Index
GGT: Gamma-Glutamyl Transferase
HDL: High-Density Lipoprotein
HOMA-IR: Homeostasis Model Assessment - Insulin Resistance
IQR: Interquartile Range
IR: Insulin Resistance
LAP: Lipid Accumulation Product
LDL: Low-Density Lipoprotein
MASLD: Metabolic Dysfunction Associated Steatotic Liver Disease
MetS: Metabolic Syndrome
MI: Myocardial Infarction
NAFLD: Non-Alcoholic Fatty Liver Disease
ROC Curve: Receiver Operating Characteristic Curve
SD: Standard Deviation
T2DM: Type 2 Diabetes Mellitus
TG/HDL-C ratio: Triglycerides To High-Density Lipoprotein Cholesterol Ratio
TIA: Transient Ischemic Attack
TyG-index: Triglycerides-Glycemia Index
VAI: Visceral Adiposity Index
VLDL: Very Low-Density Lipoprotein
