# Supplementary material for: Lipid-based insulin-resistance markers predict cardiovascular events in metabolic dysfunction associated steatotic liver disease
Source: Cardiovasc Diabetol. 2024 May 20;23:175. doi: 10.1186/s12933-024-02263-6 (PMC11106932; doi:10.1186/s12933-024-02263-6)
Supplement: Supplementary file 1 — Supplementary Material 1 [file 12933_2024_2263_MOESM1_ESM.docx]

Supplementary Table S1. MASLD patient’s characteristics according to HOMA-IR, LAP, VAI, TyG index, and TG/HDL-C Tertiles cut-offs

|  | HOMA-IR  <3.82  (n=351) | HOMA-IR  ≥3.82  (n=309) | P | LAP  <72.94  (n=229) | LAP  ≥72.94  (n=229) | P | VAI  <1.41  (n=229) | VAI  ≥1.41  (n=229) | P | TyG Index  <4.85  (n=229) | TyG Index  ≥4.85  (n=229) | P | Tg/HDL-C  <2.54  (n=229) | Tg/HDL-C  ≥2.54  (n=229) | P |
| --- | --- | --- | --- | --- | --- | --- | --- | --- | --- | --- | --- | --- | --- | --- | --- |
| Age, mean ± SD (years) | 54.6±11.7 | 55.9±11.7 | 0.154 | 55.2±12.4 | 55.2±10.8 | 0.968 | 55.5±12.6 | 55.1±11.4 | 0.730 | 54.8±12.3 | 55.7±10.7 | 0.356 | 56.6±12.2 | 54.3±11.3 | 0.013 |
| Women, n (%) | 134  (38.2) | 123  (39.8) | 0.668 | 142  (39.4) | 115  (38.1) | 0.771 | 64  (37.6) | 193  (39.4) | 0.688 | 170  (42.4) | 87  (33.6) | 0.024 | 133  (50.6) | 124  (31.2) | <0.001 |
| BMI, mean ± SD (kg/m^2^) | 28.8±4.4 | 32.1±4.9 | <0.001 | 28.7±4.4 | 32.2±4.8 | <0.001 | 29.7±5.2 | 30.6±4.8 | 0.050 | 29.9±5.0 | 31.0±4.7 | 0.005 | 29.8±5.3 | 30.6±4.6 | 0.045 |
| Obesity (BMI >30kg/m^2^), n (%) | 122  (34.8) | 193  (62.5) | <0.001 | 122  (33.9) | 193  (64.3) | <0.001 | 65  (38.2) | 250  (51.0) | 0.004 | 179  (44.6) | 136  (52.5) | 0.048 | 112  (42.6) | 203  (51.1) | 0.031 |
| Metabolic Syndrome, n (%) | 156  (44.4) | 242  (78.3) | <0.001 | 135  (37.5) | 263  (87.7) | <0.001 | 51  (30.0) | 347  (70.8) | <0.001 | 167  (41.6) | 231  (89.2) | <0.001 | 92  (35.0) | 306  (77.1) | <0.001 |
| Waist circumference, mean ± SD (cm) | 102.9±10.5 | 111.2±11.6 | <0.001 | 102.2±10.3 | 112.3±11.1 | <0.001 | 104.2±13.1 | 107.7±11.2 | 0.001 | 104.9±11.7 | 109.8±11.2 | <0.001 | 104.4±12.7 | 108.4±10.9 | <0.001 |
| Glycaemia (mg/dl), mean ± SD | 95.5±16.7 | 116.9±34.3 | <0.001 | 101.6±23.1 | 110.2±33.3 | <0.001 | 100.9±19.3 | 107.1±30.9 | 0.015 | 97.5±15.8 | 118.0±37.8 | <0.001 | 102.5±24.2 | 107.5±30.9 | 0.026 |
| Diabetes, n (%) | 56  (16.0) | 128  (41.4) | <0.001 | 87  (24.2) | 97  (32.3) | 0.020 | 34  (20.0) | 150  (30.6) | 0.008 | 75  (18.7) | 109  (42.1) | <0.001 | 64  (24.3) | 120  (30.2) | 0.098 |
| Triglycerides, median (IQR) (mg/dl) | 127.0  [95.0-172.0] | 153.0  [114.5-194.5] | <0.001 | 108.5  [87.0-127.8] | 187.5  [157.0-245.0] | <0.001 | 87.5  [71.8-101.0] | 157.0  [127.0-202.3] | <0.001 | 111.0  [88.0-134.0] | 197.0  [171.0-259.0] | <0.001 | 96.0  [81.0-113.0] | 174.0  [140.0-213.5] | <0.001 |
| HDL-C, mean ± SD (mg/dl) | 50.8±14.5 | 45.3±11.6 | <0.001 | 52.0±14.1 | 43.7±11.1 | <0.001 | 59.9±15.3 | 44.2±10.1 | <0.001 | 51.6±14.0 | 43.1±10.9 | <0.001 | 58.0±13.7 | 41.8±8.7 | <0.001 |
| Arterial hypertension, n (%) | 196  (55.8) | 210  (68.0) | 0.001 | 204  (56.7) | 202  (67.3) | 0.005 | 94  (55.3) | 312  (63.7) | 0.053 | 229  (57.1) | 177  (68.3) | 0.004 | 149  (56.7) | 257  (64.7) | 0.037 |
| Systolic BP, median (IQR) (mmHg) | 130.0  [120.0-140.0] | 130.0  [120.0-140.0] | 0.342 | 125.0  [115.0-135.0] | 130.0  [120.0-140.0] | 0.001 | 130.0  [115.0-140.0] | 130.0  [120.0-140.0] | 0.181 | 130.0  [120.0-135.0] | 130.0  [120.0-140.0] | 0.022 | 130.0  [115.0-140.0] | 130.0  [120.0-140.0] | 0.333 |
| Diastolic BP, median (IQR) (mmHg) | 80.0  [70.0-85.0] | 80.0  [70.0-85.0] | 0.118 | 80.0  [70.0-80.0] | 80.0  [75.0-85.0] | <0.001 | 80.0  [70.0-85.0] | 80.0  [70.0-85.0] | 0.105 | 80.0  [70.0-85.0] | 80.0  [75.0-85.0] | 0.024 | 89.0  [70.0-85.0] | 80.0  [75.0-85.0] | 0.044 |
| Previous CVEs, n (%) | 16  (4.6) | 18  (5.8) | 0.463 | 17  (4.7) | 17  (5.7) | 0.585 | 8  (4.7) | 25  (5.3) | 0.760 | 19  (4.7) | 15  (5.8) | 0.550 | 14  (5.3) | 20  (5.0) | 0.871 |
| AST, median (IQR) (UI/l) | 20.0  [17.0-16.0] | 22.0  [18.0-32.0] | 0.005 | 21.0  [17.0-27.8] | 22.0  [18.0-29.0] | 0.036 | 21.0  [17.0-26.0] | 21.5  [18.0-29.0] | 0.197 | 21.0  [17.0-28.0] | 22.0  [18.0-29.0] | 0.035 | 20.0  [16.0-26.0] | 22.0  [18.0-30.0] | 0.003 |
| ALT, median (IQR) (UI/l) | 23.0  [12.0-39.0] | 31.0  [23.0-46.0] | <0.001 | 26.0  [18.0-41.5] | 28.5  [21.0-43.0] | 0.026 | 26.0  [18.0-40.0] | 28.0  [20.0-43.0] | 0.096 | 26.0  [18.0-40.0] | 28.0  [21.0-43.0] | 0.010 | 24.0  [17.0-37.0] | 29.0  [21.0-44.0] | <0.001 |
| GGT, median (IQR) (UI/l) | 23.0  [16.0-35.3] | 29.5  [20.0-45.0] | <0.001 | 23.0  [16.0-37.0] | 30.0  [20.0-43.8] | <0.001 | 21.0  [15.0-36.0] | 28.0  [18.0-42.0] | 0.001 | 23.0  [16.0-36.0] | 31.0  [21.0-47.0] | <0.001 | 21.0  [15.0-37.0] | 29.0  [20.0-42.0] | <0.001 |
| Platelets, mean ± SD (x10^9^/l) | 241.1±65.3 | 234.8±63.1 | 0.209 | 236.5±66.5 | 240.1±61.6 | 0.476 | 227.4±53.2 | 241.9±67.4 | 0.011 | 237.4±64.3 | 239.4±64.4 | 0.685 | 237.1±65.9 | 238.9±63.3 | 0.718 |
| High Fib-4, n (%) | 7 (2.0) | 8  (2.6) | 0.609 | 7  (1.9) | 7  (2.7) | 0.535 | 5  (2.9) | 10  (2.0) | 0.497 | 6  (1.5) | 9  (3.5) | 0.096 | 6  (2.3) | 9  (2.3) | 0.990 |
| Low Fib-4, n (%) | 296  (84.3) | 243  (78.6) | 0.059 | 301  (83.6) | 238  (79.3) | 0.157 | 135  (79.4) | 404  (82.4) | 0.378 | 332  (82.8) | 207  (79.9) | 0.352 | 216  (82.1) | 323  (81.4) | 0.803 |
| Incident CVEs, n (%) | 20  (5.7) | 33  (10.7) | 0.019 | 18  (5.0) | 35  (11.7) | 0.002 | 4  (2.4) | 49  (10.0) | 0.002 | 18  (4.5) | 35  (13.5) | <0.001 | 9  (3.4) | 44  (11.1) | <0.001 |

Abbreviations: ALT – Alanine Aminotransferase, AST – Aspartate Aminotransferase, BMI – Body Mass Index, CVEs – Cardiovascular Events, GGT – Gamma-Glutamyl Transferase, HDL-C – High-Density Lipoprotein Cholesterol, BP – Blood Pressure, LAP – Lipid Accumulation Product, VAI – Visceral Adiposity Index, TyG Index – Triglyceride-Glucose Index, TG/HDL-C – Triglycerides to HDL-cholesterol ratio; MASLD – Metabolic Dysfunction-Associated Steatotic Liver Disease, IQR – Interquartile Range, SD – Standard Deviation;

Supplementary Table S2. Association between insulin resistance markers and MASLD diagnosis, according to diabetes.

|  | With Diabetes  OR (95%CI)° | Without Diabetes  OR (95%CI)° | p for interaction |
| --- | --- | --- | --- |
| HOMA-IR | 3.37 (1.00-11.42) | 7.31 (4.03-13.26) | 0.290 |
| LAP | 6.69 (1.67-26.73) | 8.23 (4.34-15.59) | 0.577 |
| VAI | 1.83 (0.58-5.72) | 5.42 (3.21-9.15) | 0.134 |
| TyG Index | 1.83 (0.54-6.19) | 5.30 (3.04-9.24) | 0.076 |
| TG/HDL-C | 1.86 (0.56-6.16) | 4.88 (2.83-8.40) | 0.111 |

°Adjusted for: age, sex, obesity, blood hypertension, previous cardiovascular events (CVEs), and low fibrosis 4 (Fib4). Abbreviations: aHR: adjusted Hazard Ratio; CI: coincidence interval; HOMA-IR: Homeostasis Model A for Insulin Resistance; LAP: Lipid Accumulation Product; VAI: Visceral Adiposity Index; TyG index: Triglycerides-Glycaemia Index; TG/HDL-C: Triglycerides to High-Density Lipoprotein ratio.

Supplementary Table S3. Association between insulin resistance markers and risk of cardiovascular events in MASLD patients, according to previous cardiovascular events and diabetes.

|  | Previous CVEs  HR° (95%CI) | No previous CVEs  HR° (95%CI) | p _for interaction_ | With Diabetes  HR (95%CI)^#^ | Without Diabetes  HR (95%CI) ^#^ | p _for interaction_ |
| --- | --- | --- | --- | --- | --- | --- |
| HOMA-IR | 3.25 (0.59-17.80) | 1.34 (0.65-2.73) | 0.470 | 1.41 (0.46-4.35) | 1.80 (0.84-3.84) | 0.943 |
| LAP | 1.95 (0.54-7.04) | 2.52 (1.12-4.52) | 0.862 | 3.19 (1.16-9.75) | 2.03 (0.93-4.45) | 0.272 |
| VAI | 1.25 (0.25-6.16) | 6.49 (1.55-27.18) | 0.267 | N/A* | N/A* | N/A* |
| TyG Index | 1.95 (0.51-7.56) | 2.77 (1.34-5.62) | 0.334 | 2.70 (0.98-7.44) | 2.43 (1.14-5.17) | 0.758 |
| TG/HDL-C | 3.19 (0.61-16.76) | 2.94 (1.22-7.10) | 0.646 | 5.51 (1.25-24.23) | 2.20 (0.91-5.30) | 0.256 |

°Adjusted for: age, sex, obesity, blood hypertension, diabetes, low fibrosis 4 (Fib4); ^#^ Adjusted for: age, sex, obesity, blood hypertension, previous cardiovascular events (CVEs), low fibrosis 4 (Fib4).

*In diabetic patients, all patients developing CVEs had VAI values above the cut-off. Abbreviations: aHR: adjusted Hazard Ratio; CI: coincidence interval; HOMA-IR: Homeostasis Model A for Insulin Resistance; LAP: Lipid Accumulation Product; VAI: Visceral Adiposity Index; TyG index: Triglycerides-Glycaemia Index; TG/HDL-C: Triglycerides to High Density Lipoprotein ratio.
